# Supplementary material for: Mutant Lef1 controls Gata6 in sebaceous gland development and cancer
Source: EMBO J. 2019 Mar 18;38(9):e100526. doi: 10.15252/embj.2018100526 (PMC6484415; doi:10.15252/embj.2018100526)

Appendix Figures

Appendix Figure S1: Reduction in Plet1 and Atp6v1c2 expression on loss of Gata6 in K14ΔNLef1 tumors.

Appendix Figure S2: Reduction in Mlh1 and Msh2 expression on loss of Gata6 in K14ΔNLef1 tumors.

Appendix Figure S3: Gata6 expression in a range of human skin tumors.

Appendix Figure legends

Appendix Figure S1: Reduction in Plet1 and Atp6v1c2 expression on loss of Gata6 in K14ΔNLef1 tumors.

Digital tissue microarray of WT, K14ΔNLef1 and K14ΔNLef1:cKO mouse skin tumor sections labelled with antibodies to Plet1 and Atp6v1c2. Some of these images are also depicted in Fig 4D. Scale bar: 100 µm.

Appendix Figure S2: Reduction in Mlh1 and Msh2 expression on loss of Gata6 in K14ΔNLef1 tumors.

(**A**) Digital tissue microarray of K14ΔNLef1 and K14ΔNLef1:cKO mice skin tumor sections labelled with Mlh1 antibody. Technical controls are displayed (without primary antibody incubation). A positive control (UVB-irradiated WT mice) and a negative control (WT mice) are also included. Some of these images are also depicted in Fig 5D.

(**B**) Digital tissue microarray of K14ΔNLef1 and K14ΔNLef1:cKO mice skin tumor sections labelled with Msh2 antibody with similar controls as in (A). Some of these images are also depicted in Fig 5E.

Data information: (**A and B**) Scale bar: 250 µm.

Appendix Figure S3: Gata6 expression in a range of human skin tumors.

Digital tissue microarray of human skin tumors sections labelled with Gata6 and PanKeratin antibodies. Some of these images are also depicted in Fig 6B. Scale bar: 100 µm.

Appendix Figure S1

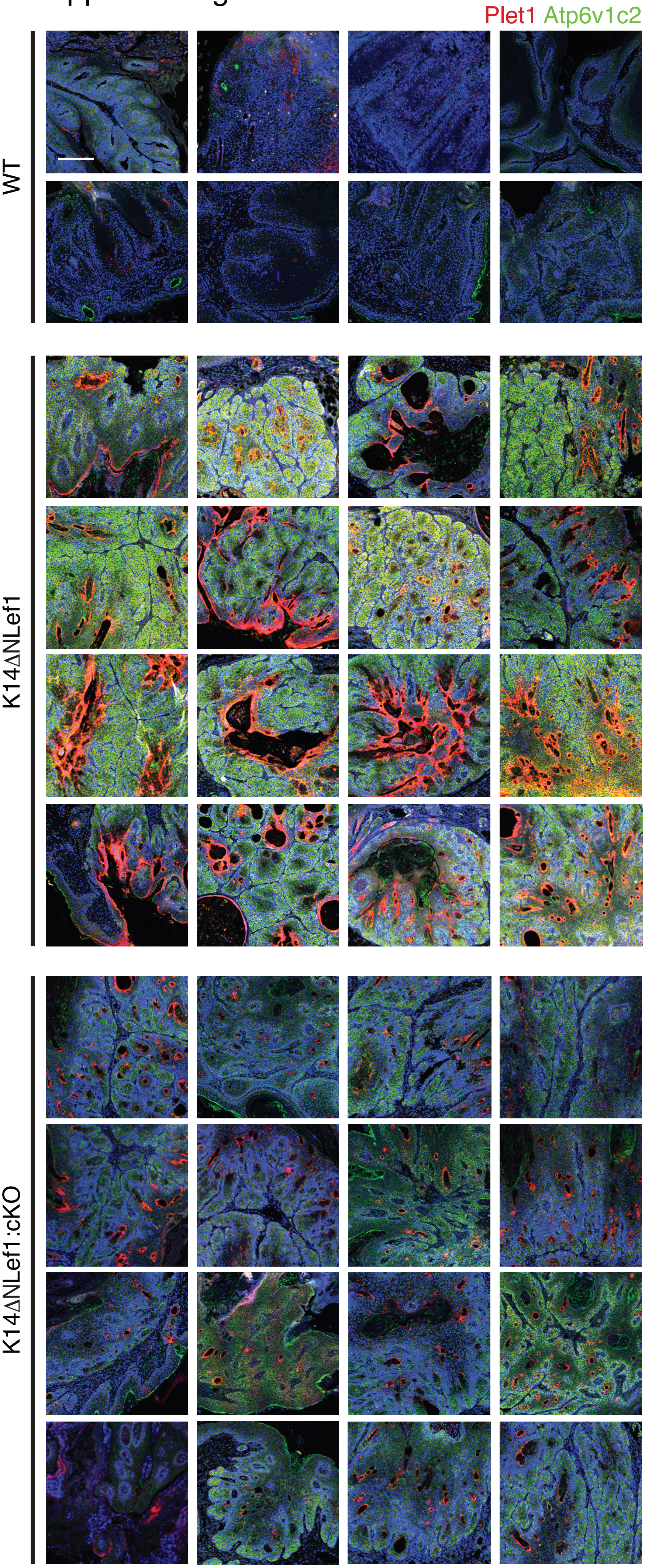

Appendix Figure S2

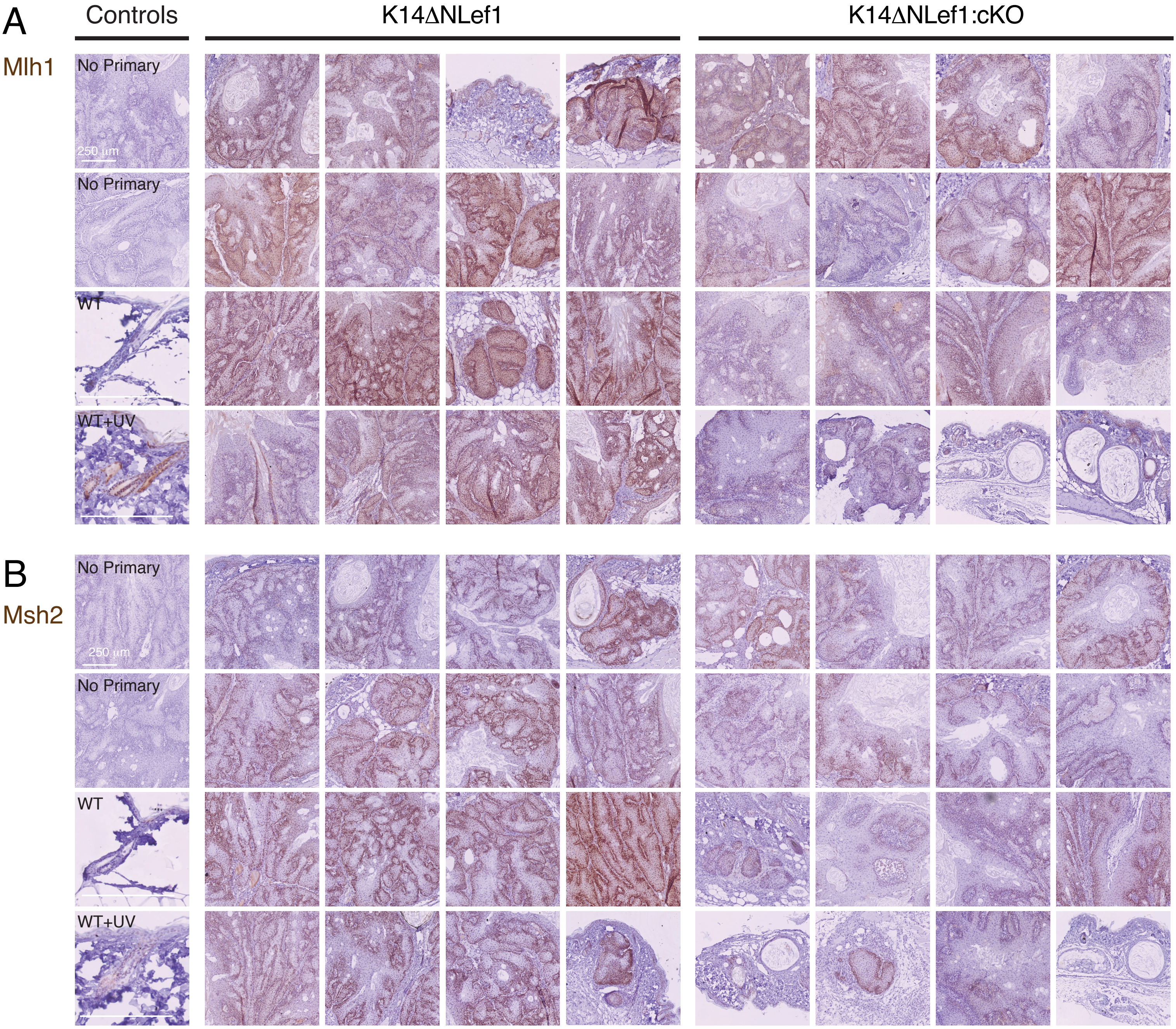

Appendix Figure S3

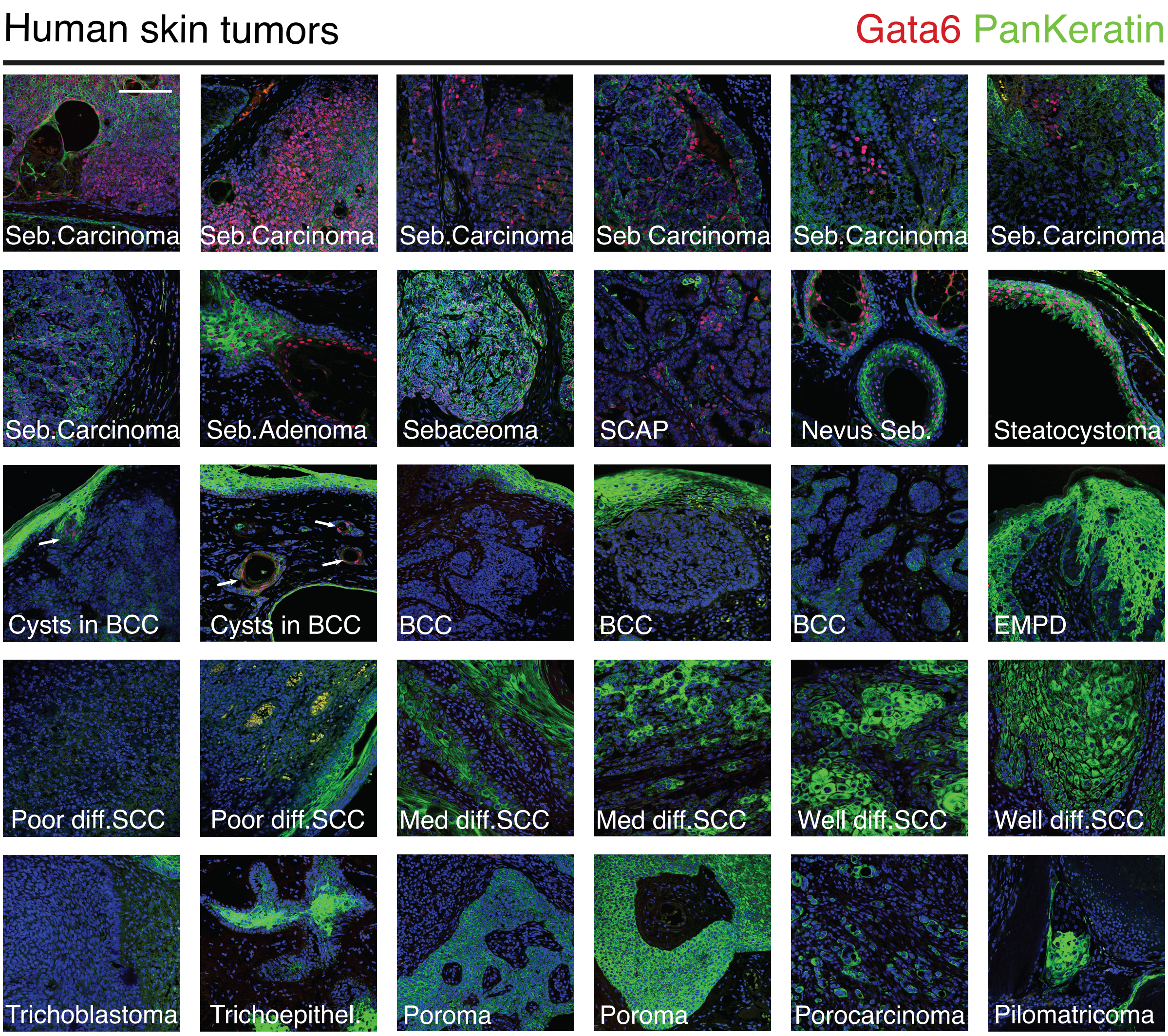

Supplement: Supplementary file 1 — Appendix [file EMBJ-38-e100526-s001.pdf]
