# Supplementary material for: Mutant Lef1 controls Gata6 in sebaceous gland development and cancer
Source: EMBO J. 2019 Mar 18;38(9):e100526. doi: 10.15252/embj.2018100526 (PMC6484415; doi:10.15252/embj.2018100526)
Supplement: Supplementary file 2 — Expanded View Figures PDF [file EMBJ-38-e100526-s002.pdf]

Expanded View Figures

Figure EV1. Validation of transcriptional changes observed in K14ΔNLef1 keratinocytes.

A Flow-sorting strategy for basal keratinocytes ($Itga6^{+}Cd34^{-}$) and bulge stem cells HFSC ($Itga6^{+}Cd34^{+}$).

B *Z*-score heat-map representing DEG between WT- or K14ΔNLef1-sorted all, basal or HFSC keratinocytes. Validation of these results was performed by RT–qPCR. Data are means ± SEM from 3 to 4 mice. (ns) Not significant; **P* < 0.05; ***P* < 0.005; ****P* < 0.0005; *****P* < 0.00005; ordinary one-way ANOVA.

C Gene Ontology enrichment analysis and network visualization of DEG in HF vs IFE vs. SG (Donati *et al*, 2017) and WT vs K14ΔNLef1 keratinocyte expression profiles.

D *Z*-score heat-map representing the intersection between ΔNLef1 direct target genes (ChIP-Seq signals ± 3 kb around the peaks centers (C); right panels), DEG in SG vs. IFE and HF (Donati *et al*, 2017), and WT vs. K14ΔNLef1 keratinocyte expression profiles (obtained in Fig 1I; left panels).

Data information: (B–D) Upregulated genes in K14ΔNLef1 epidermis are shown in red and downregulated genes in blue.

© 2019 The Authors

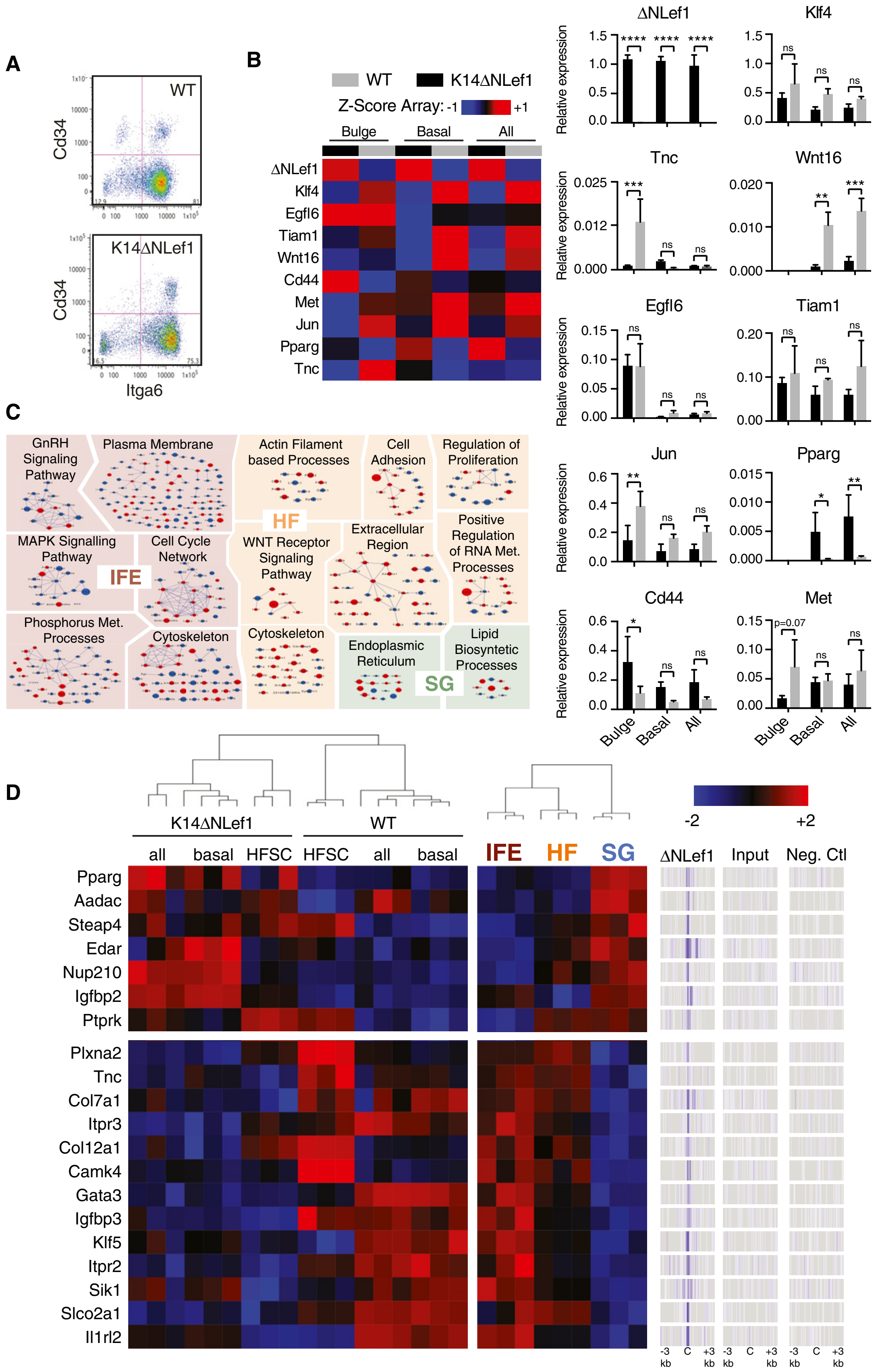
A
WT
Cd34
K14ΔNLef1
Itga6
B
WT
K14ΔNLef1
Z-Score Array: -1 +1
Bulge
Basal
All
ΔNLef1
Klf4
Egfl6
Tiam1
Wnt16
Cd44
Met
Jun
Pparg
Tnc
Relative expression
C
GnRH Signaling Pathway
Plasma Membrane
Actin Filament based Processes
Cell Adhesion
Regulation of Proliferation
HF
MAPK Signalling Pathway
Cell Cycle Network
IFE
WNT Receptor Signaling Pathway
Extracellular Region
Positive Regulation of RNA Met. Processes
Phosphorus Met. Processes
Cytoskeleton
Cytoskeleton
Endoplasmic Reticulum
SG
Lipid Biosyntetic Processes
D
K14ΔNLef1
WT
all
basal
HFSC
HFSC
all
basal
IFE
HF
SG
-2
+2
ΔNLef1
Input
Neg. Ctl
Pparg
Aadac
Steap4
Edar
Nup210
Igfbp2
Ptprk
Plxna2
Tnc
Col7a1
Itpr3
Col12a1
Camk4
Gata3
Igfbp3
Klf5
Itpr2
Sik1
Slco2a1
Il1rl2
-3 kb
C
+3 kb

Figure EV1.

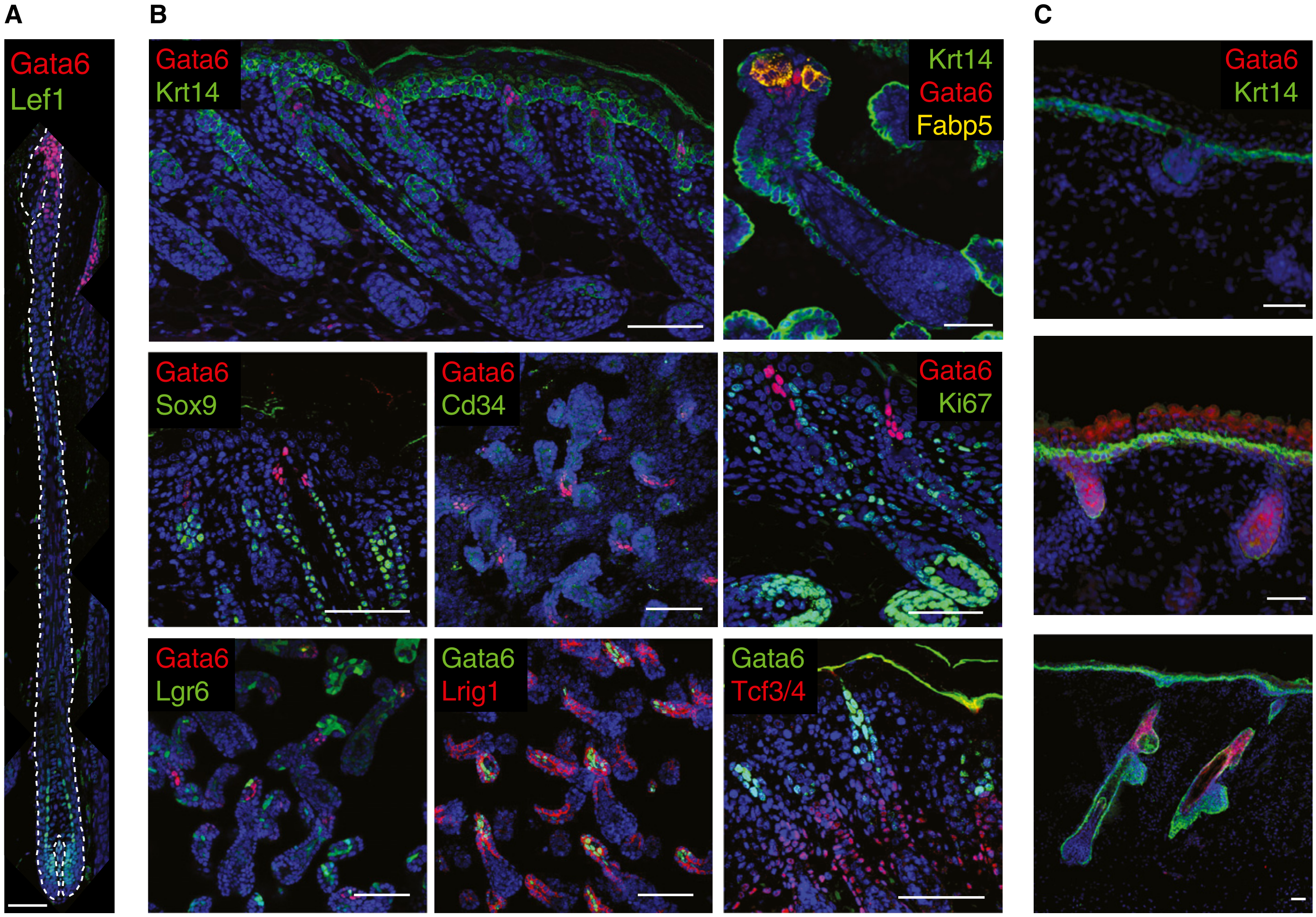

Figure EV2. Gata6 expression in SD/SG compartment.

A Adult mouse dorsal skin in late anagen stained for Gata6 and Lef1 and counterstained with Dapi.

B Whole-mount tail epidermis or dorsal skin sections of P1 WT mice were stained for Gata6 and Krt14, Fabp5, Ki67, several HF markers (Lrig1, Cd34, Sox9), and Tcf3/4. Lgr6 cells (GFP^{+}) are shown in whole-mount tail epidermis from P1 Lgr6EGFPCreERT2 mice.

C Sections of human embryonic skin at different HF stages stained for Gata6 and Krt14, and counterstained with Dapi.

Data information: (A–C) Scale bars: 50 μm.

 © 2019 The Authors

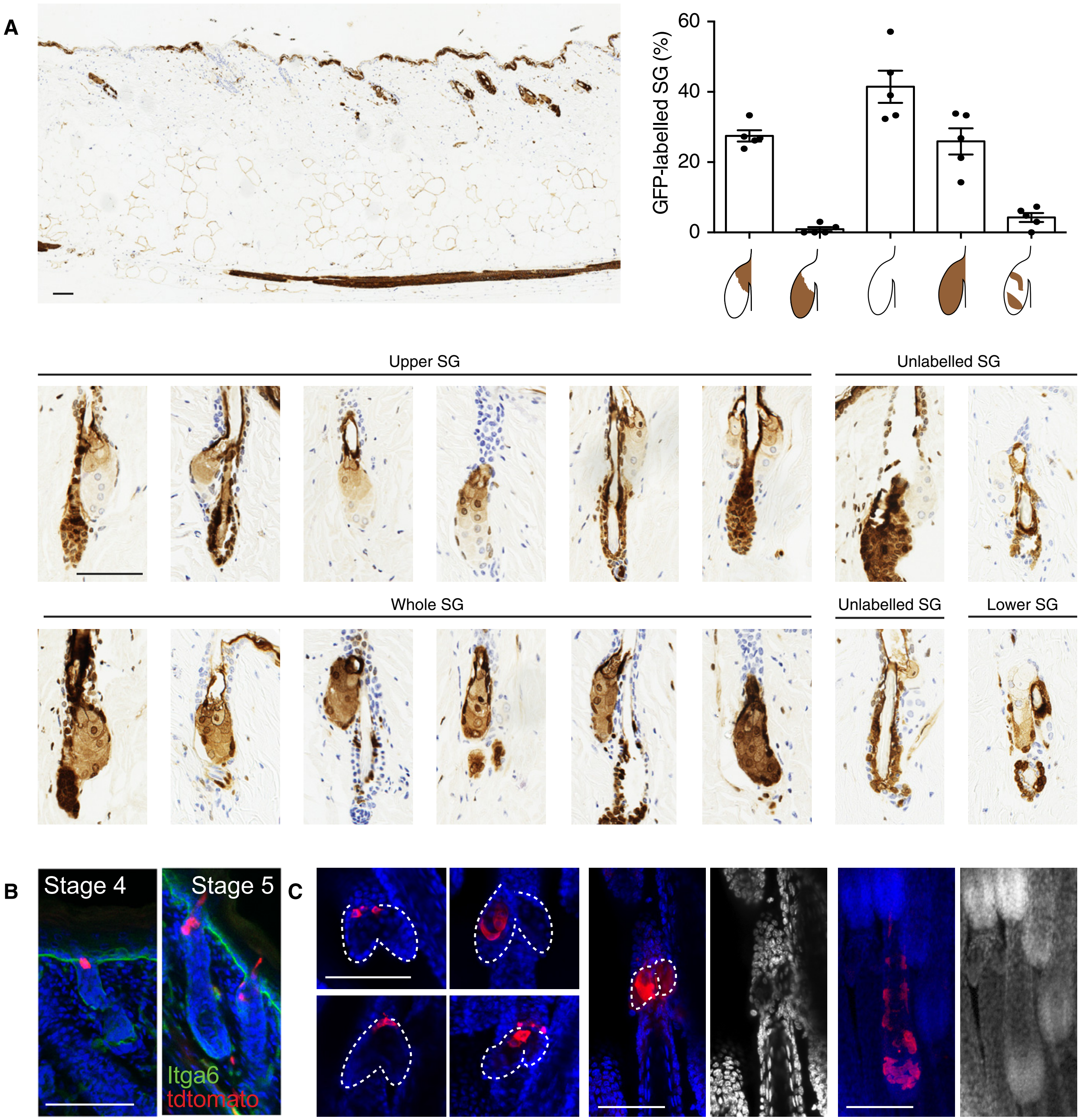

Figure EV3. Mechanisms of SG development.

A Representative skin section stained for GFP (brown) to reveal chimerism in WT:GFP mice (large upper panel). Small images highlight different stained SG. Nuclei are counterstained with hematoxylin. Quantification of the percentage of SG with different contributions of GFP labeled cells as depicted below the graph. Experiment was performed in five independent mice. Data are means ± SEM.

B Lineage tracing experiments as in Fig 3D. Representative example of tdTomato-labeled cells counterstained with Dapi at 2 days after recombination.

C Representative examples of whole-mount epidermis positive for tdTomato and counterstained with Dapi showing staining of the upper SG (left panels), or exceptionally the lower SG (middle panel) or the bottom of the HF (right panel).

Data information: (A–C) Scale bars: 50 μm.

© 2019 The Authors

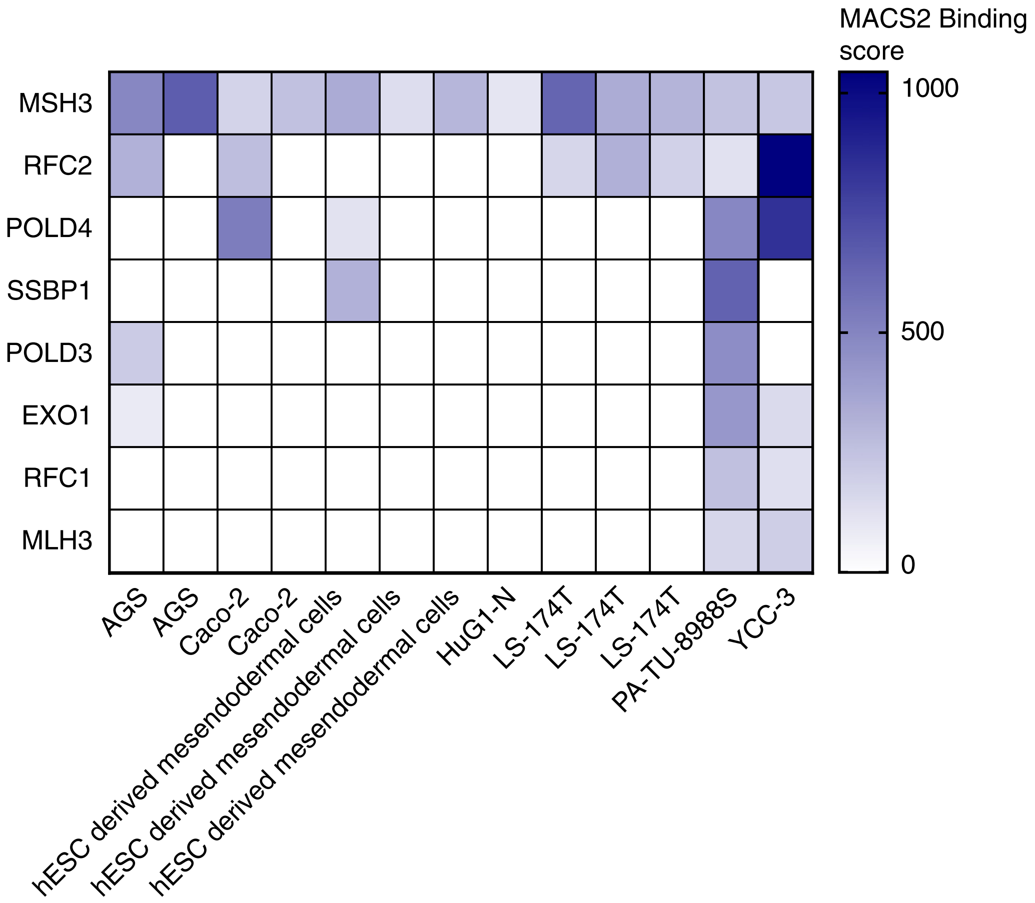

Figure EV4. Direct targets of human Gata6 predicted by ChIP-Atlas.

Human Gata6 ChIP-Seq datasets were interrogated using the "Target Genes" module of ChIP-Atlas (Oki *et al*, 2018 and http://chip-atlas.org). The included datasets were obtained from human ES cell-derived mesendodermal cells (GEO accession numbers: GSM1505658, GSM1505660, GSM1505662) and human adenocarcinoma cell lines from stomach (AGS (GSM1250897, GSM1255511), HuG1-N (GSM1255513), YCC-3 (GSM1250893)), colon (Caco-2 (GSM575226, GSM575227), LS-174T (GSM1197320, GSM1197321, GSM1197322), and pancreas (PA-TU-8988S (GSM1151694)). A Gata6-binding peak was found within 10 kb from the transcription start in at least two different datasets for eight genes of the MMR pathway (Msh3, Rfc2, Pold4, Ssbp1, Pold3, Exo1, Rfc1, and Mlh3). The heat-map represents MACS2 binding scores for the MMR genes in the corresponding datasets.

© 2019 The Authors
